# Development of new scores for atherosclerotic cardiovascular disease using specific medical examination items: the Suita Study

**DOI:** 10.1265/ehpm.23-00099

**Published:** 2023-10-27

**Authors:** Ahmed Arafa, Rena Kashima, Yuka Yasui, Haruna Kawachi, Chisa Matsumoto, Saya Nosaka, Masayuki Teramoto, Miki Matsuo, Yoshihiro Kokubo

**Affiliations:** 1 Department of Preventive Cardiology, National Cerebral and Cardiovascular Center, Suita, Japan; 2 Department of Public Health, Faculty of Medicine, Beni-Suef University, Beni-Suef, Egypt; 3 Department of Cardiovascular Pathophysiology and Therapeutics, Graduate School of Medicine, Osaka University, Suita, Japan; 4 Graduate School of Human Life and Science, Doshisha Women’s College of Liberal Arts, Kyoto, Japan; 5 Department of Environmental Medicine and Population Sciences, Graduate School of Medicine, Osaka University, Suita, Japan; 6 Department of Cardiology, Center for Health Surveillance and Preventive Medicine, Tokyo Medical University Hospital, Shinjuku, Japan; 7 Department of Hypertension and Nephrology, National Cerebral and Cardiovascular Center, Suita, Japan

**Keywords:** Risk scores, Prediction models, Stroke, Coronary heart disease

## Abstract

**Background:**

We previously developed risk models predicting stroke, coronary heart disease (CHD), and cardiovascular disease (CVD) among Japanese people from the Suita Study. Yet, applying these models at the national level was challenging because some of the included risk factors differed from those collected in the Japanese governmental health check-ups, such as *Tokutei-Kenshin*. We, therefore, conducted this study to develop new risk models for stroke, CHD, and atherosclerotic CVD (ASCVD), based on data from the Suita Study. The new models used traditional cardiovascular risk factors similar to those in the Japanese governmental health check-ups.

**Methods:**

We included 7,413 participants, aged 30–84 years, initially free from stroke and CHD. All participants received baseline health examinations, including a questionnaire assessing their lifestyle and medical history, medical examination, and blood and urine analysis. The risk factors of stroke, CHD, and ASCVD (cerebral infarction or CHD) were determined using the multivariable-adjusted Cox regression. The models’ performance was assessed using the C-statistics for discrimination and the Hosmer-Lemeshow for calibration. We also developed three simple scores (zero to 100) that could predict the 10-year incidence of stroke, CHD, and ASCVD.

**Results:**

Within 110,428 person-years (median follow-up = 16.6 years), 410 stroke events, 288 CHD events, and 527 ASCVD events were diagnosed. Age, smoking, hypertension, and diabetes were associated with stroke, CHD, and ASCVD risk. Men and those with decreased high-density lipoproteins or increased low-density lipoproteins showed a higher risk of CHD and ASCVD. Urinary proteins were associated with an increased risk of stroke and ASCVD. The C-statistic values of the risk models were >0.750 and the p-values of goodness-of-fit were >0.30. The 10-year incidence of stroke, CVD, and ASCVD events was 3.8%, 3.5%, and 5.7% for scores 45–54, 10.3%, 11.8%, and 19.6% for scores 65–74, and 27.7%, 23.5%, and 60.5% for scores ≥85, respectively.

**Conclusions:**

We developed new Suita risk models for stroke, CHD, and ASCVD using variables similar to those in the Japanese governmental health check-ups. We also developed new risk scores to predict incident stroke, CHD, and ASCVD within 10 years.

**Supplementary information:**

The online version contains supplementary material available at https://doi.org/10.1265/ehpm.23-00099.

## 1. Introduction

Cardiovascular disease (CVD), especially stroke and coronary heart disease (CHD), remains the leading cause of morbidity and mortality in Japan and globally [1]. Determining people at elevated risk of developing CVD is essential for risk prevention [2, 3].

In this context, we previously developed risk models predicting stroke [4], CHD [5], and CVD [6] using data from the Suita Study, a prospective cohort study representing the general population in urban Japan. Despite being used for health counseling in many Japanese health settings, applying the Suita risk models at the national level was challenging for two main reasons. First, some included risk factors, such as arrhythmia, cardiac murmurs, and chronic kidney disease (CKD), are not regularly assessed in the health check-ups conducted in local municipalities by the Japanese Government, such as *Tokutei-Kenshin*. In contrast, other risk factors, such as urinary proteins, are regularly assessed in these health check-ups but were not included in the Suita risk models [7], because CKD was used instead. Second, previous Suita risk models utilized different categorizations and scoring systems [4–6], making it difficult to compare them and inconvenient for practical application.

Hence, based on data from the Suita Study, we developed new risk models and scores for stroke, CHD, and atherosclerotic CVD (ASCVD) using risk factors similar to those that are regularly assessed in Japanese governmental health check-ups.

## 2. Methods

### 2.1. Participants

The Suita population (n = 8,360) included two cohorts randomly selected by sex and 10-year age category from the urban city of Suita (recruited between 1989 and 1998) and a volunteer group (recruited between 1992 and 2006). The first cohort included 12,200 participants (6,485 participants were eligible), the second cohort included 3,000 participants (1,329 participants were eligible), and the volunteer group included 546 participants. The baseline assessment was held at the National Cerebral Cardiovascular Centre (NCVC) in Suita. It included data collection about lifestyle, blood and urine sampling, clinical and fundus examinations, and electrocardiography (ECG). Participants were encouraged to return every two years for follow-up. In this study, we excluded participants who had a positive history of stroke or CHD (n = 367), lacked baseline data about weight, height, waist circumference (WC), smoking behavior, alcohol consumption, blood pressure (BP), blood glucose, low- or high-density lipoprotein-cholesterol (LDL-c or HDL-c), or urinary proteins (n = 131), or were lost to follow-up (n = 449). Eventually, 7,413 participants, aged 30–84 years, were included for analysis. All participants were followed up from the date of baseline assessment until the date of stroke or CHD event, death, leaving the study, or the end of follow-up (December 31, 2013), whichever came first.

### 2.2. Assessment of outcomes

The health status of participants was examined during the follow-up check-ups conducted every two years in addition to the annual follow-ups in the form of self-administered questionnaires sent by mail or telephone interviews. Besides, registered physicians, who were blind to participants’ baseline data, revised their medical records. Stroke was diagnosed per the US National Survey of Stroke criteria based on computed tomographic scans and magnetic resonance images [8]. CHD included myocardial infarctions, defined per the criteria of the World Health Organization Multinational MONItoring of Trends and Determinants in CArdiovascular Disease (WHO MONICA) Project [9], percutaneous coronary intervention, coronary artery bypass grafting, and sudden cardiac death within 24 hours. ASCVD included cerebral infarction and CHD.

### 2.3. Assessment of risk factors

Data about risk factors were collected during the baseline examination. Lifestyle factors, such as smoking and alcohol consumption, and clinical histories were self-reported. Well-trained nurses revised the health and medical records of participants. Weight and height were measured for body mass index (BMI) calculation (weight in Kg/height in m^2^). WC was measured in a standing position at the umbilical level to the nearest one cm without skin compression after instructing participants to breathe out. Participants wore light clothes for weight and WC assessment. BP was measured three times and the average of the last two measurements was used for analysis. Blood samples were collected and centrifuged before routine blood examinations, including blood glucose, LDL-c, HDL-c, and creatinine. Urine samples were also collected and analyzed. Besides, participants underwent a standard 12-lead ECG examination for three minutes. After revising the *Tokutei-Kenshin* questionnaire [7], previous Suita CVD risk models [4–6], and Japanese guidelines for obesity, hypertension, diabetes, and dyslipidemia [10–12], the following variables were included: sex, age (30–39, 40–44, 45–49, 50–54, 55–59, 60–64, 65–69, 70–74, and ≥75 years), BMI: (<18.5, 18.5–24.9, or ≥25 kg/m^2^), WC (<85 cm in men / <90 cm in women [normal] or ≥85 cm in men / ≥90 cm in women [high]), smoking (never, former, or current), alcohol consumption (never, former, or current), BP (<120/80 mmHg [optimal BP], 120–139/80–89 mmHg [high-normal or elevated BP], or ≥140/90 mmHg / receiving medications [hypertension]), blood glucose (fasting blood glucose (FBG) <100 mg/dL / HbA1c ≤5.5% [normal], FBG 100–125 mg/dL / HbA1c 5.6–6.4% [impaired], or FBG ≥126 mg/dL / HbA1c ≥6.5% / receiving medication [diabetes]), LDL-c (<100, 100–159 or ≥160 mg/dL), HDL-c (<40 or ≥40 mg/dL), and urinary proteins (− or ≥+1). In further analyses, we added variables that have been performed in the Japanese governmental health check-ups for high-risk individuals only. These variables included CKD, defined as an estimated glomerular filtration rate <60 mL/min/1.73 m^2^ (yes or no), hypertensive retinopathy diagnosed using the Keith–Wagener–Barker classification [13], and ECG findings per the Minnesota codes for atrioventricular conduction defect (6-codes), high-amplitude R (3-codes), ST-T abnormalities (4-codes and 5-codes), and atrial fibrillation or flutter (8-3-1 to 4) (yes or no, each).

### 2.4. Statistical analysis

**Fig. 1:**
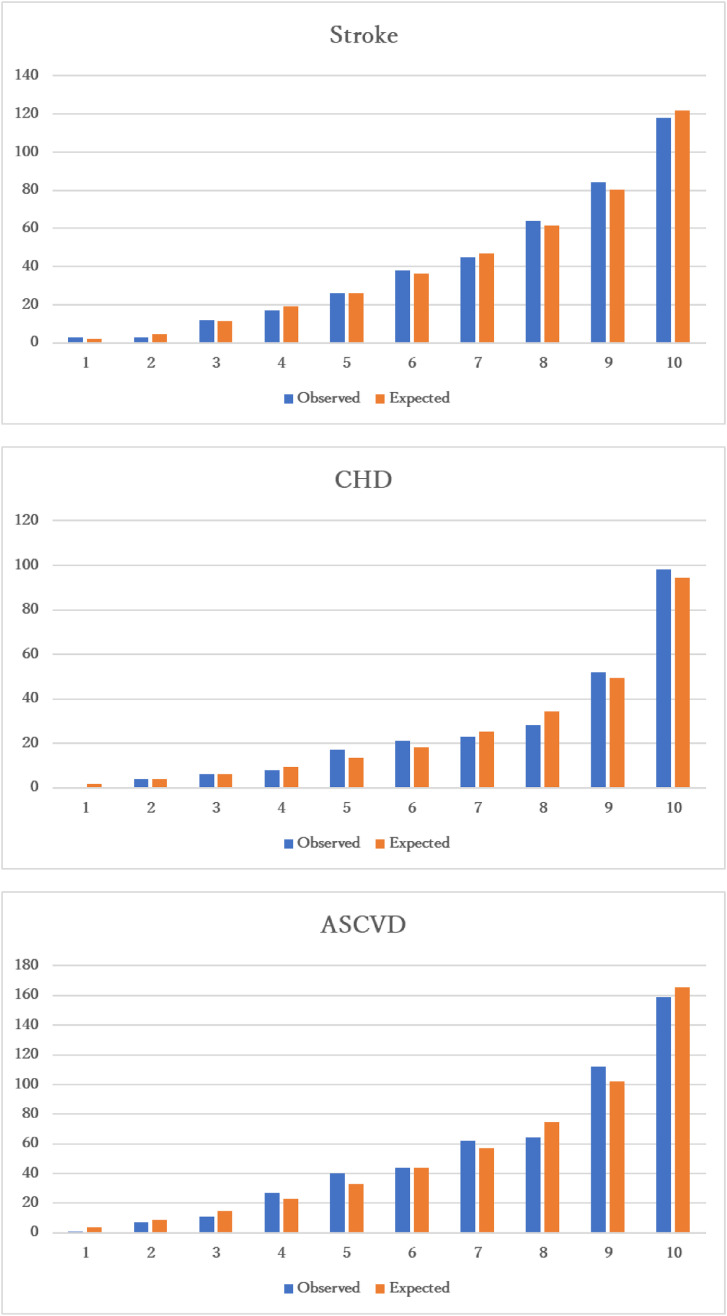
Hosmer-Lameshow goodness-of-fit test P-values of goodness-of-fit were 0.981 for stroke, 0.746 for coronary heart disease (CHD), and 0.326 for atherosclerotic cardiovascular disease (ASCVD)

First, we described the proportions of the baseline personal and clinical characteristics of participants by the outcome: no events, stroke, CHD, and ASCVD (Table 1). Then, we conducted age-and sex-adjusted Cox proportional hazard models to detect potential risk factors for stroke, CHD, and ASCVD. Among participants who developed both cerebral infarction and CHD, the follow-up in the ASCVD analysis was censored when the earlier event occurred. Factors that showed statistically significant associations in the age-and sex-adjusted models (p < 0.10) were included in multivariable-adjusted models, and variables that showed statistically significant associations in the later models (p < 0.05) were considered the final risk models (Table 2). Then, weights were assigned based on the β-coefficient of significant variables in the final risk model, and these weights were translated into risk scores ranging between zero and 100 (Table 3). We tested the final risk models in terms of discriminative ability using the C-statistics (Table 4) [14] and calibration using the Hosmer-Lemeshow statistics by comparing the observed and expected incident events by decile of risk (Fig. 1) [15]. We also calculated the 10-year incidence of stroke, CHD, and ASCVD by their risk scores (Table 5). The interaction of sex in the association between other potential risk factors and stroke, CHD, and ASCVD was examined as well (Supplementary Table 1). We repeated the analysis after adding the following variables one by one: CKD, hypertensive retinopathy, atrioventricular conduction defect, high-amplitude R, ST-T abnormalities, and atrial fibrillation or flutter (Supplementary Table 2). SAS version 9.4 software (SAS Institute Inc, Cary, NC) was used for statistical analysis.

**Table 1:** Traditional cardiovascular disease risk factors at baseline

**Risk factors**		**No events**	**Stroke**	**Coronary heart disease**	**Atherosclerotic cardiovascular disease**
Number of participants		6,715	410	288	527
Sex, %	Men	45.6	54.9	67.0	63.2
	Women	54.4	45.1	33.0	36.8
Age (years), %	30–39	16.9	1.2	3.5	2.1
	40–44	10.0	2.7	3.8	3.0
	45–49	10.0	5.1	6.3	5.3
	50–54	11.7	7.3	9.0	8.5
	55–59	12.0	12.2	11.8	11.8
	60–64	13.7	17.1	21.5	21.1
	65–69	9.6	19.0	16.3	18.0
	70–74	10.0	21.0	17.4	18.8
	≥75	6.1	14.4	10.4	11.4
Body mass index (kg/m^2^), %	<18.5	8.5	6.8	3.8	4.7
	18.5–24.9	72.1	68.3	69.1	69.1
	≥25	19.4	24.9	27.1	26.2
Waist circumference (cm), %	Normal	74.7	62.7	61.8	60.3
	High	25.3	37.3	38.2	39.7
Smoking, %	Never	55.0	46.6	38.5	40.4
	Former	15.7	20.0	25.7	24.5
	Current	29.3	33.4	35.8	35.1
Alcohol drinking, %	Never	45.5	42.9	47.2	44.4
	Former	2.3	3.7	3.5	3.2
	Current	52.2	53.4	49.3	52.4
Blood pressure (mmHg), %	Optimal	38.0	17.8	13.9	14.2
	High-normal	32.7	27.8	29.2	28.1
	Hypertension	29.3	54.4	56.9	57.7
Blood glucose (mg/dL), %	Normal	67.7	51.7	46.5	48.2
	Impaired	27.5	36.8	41.3	38.9
	Diabetes	4.8	11.5	12.2	12.9
High-density lipoprotein-cholesterol (mg/dL), %	<40	13.4	18.0	27.1	24.3
	≥40	86.6	82.0	72.9	75.7
Low-density lipoprotein-cholesterol (mg/dL), %	<100	20.2	17.1	10.8	14.6
	100–159	62.8	63.9	57.6	60.0
	≥160	17.0	19.0	31.6	25.4
Urinary proteins, %	−	82.5	72.2	77.4	75.3
	≥+1	17.5	27.8	22.6	24.7

**Table 2:** Risk models of stroke, coronary heart disease, and atherosclerotic cardiovascular disease

**Risk factors**		**Stroke**		**Coronary heart disease**		**Atherosclerotic ** **cardiovascular disease**	

		**β1**	**β2**	**β1**	**β2**	**β1**	**β2**
Sex	Men	0.419*	0.107	0.921*	0.911*	0.742*	0.429*
	Women	Ref	Ref	Ref	Ref	Ref	Ref
Age (years)	30–39	Ref	Ref	Ref	Ref	Ref	Ref
	40–44	1.186*	1.131*	0.507*	0.317	0.775*	0.619*
	45–49	1.842*	1.773*	1.025*	0.779*	1.344*	1.151*
	50–54	2.055*	1.858*	1.230*	0.778*	1.661*	1.275*
	55–59	2.630*	2.424*	1.583*	1.016*	2.052*	1.625*
	60–64	2.865*	2.581*	2.050*	1.344*	2.500*	1.967*
	65–69	3.418*	3.143*	2.259*	1.533*	2.803*	2.263*
	70–74	3.674*	3.417*	2.483*	1.763*	2.995*	2.483*
	≥75	4.050*	3.768*	2.761*	2.002*	3.238*	2.695*
Body mass index (Kg/m^2^)	<18.5	−0.075	0.024	−0.590*	−0.297	−0.397*	−0.147
	18.5–24.9	Ref	Ref	Ref	Ref	Ref	Ref
	≥25	0.268*	0.010	0.350*	0.104	0.308*	0.020
Waist circumference	Normal	Ref	Ref	Ref	Ref	Ref	Ref
	High	0.311*	0.171	0.239*	−0.125	0.328*	0.069
Smoking	Never	Ref	Ref	Ref	Ref	Ref	Ref
	Former	0.088	0.077	0.197	0.233	0.222	0.197
	Current	0.460*	0.527*	0.330*	0.471*	0.409*	0.474*
Alcohol drinking	Never	Ref	Ref	Ref	Ref	Ref	Ref
	Former	0.310	—	−0.139	−0.268	−0.078	—
	Current	0.169	—	−0.431*	−0.465*	−0.152	—
Blood pressure (mmHg)	Optimal	Ref	Ref	Ref	Ref	Ref	Ref
	High-normal	0.152	0.134	0.547*	0.535*	0.424*	0.390*
	Hypertension	0.707*	0.632*	1.203*	1.179*	1.067*	0.987*
Blood glucose (mg/dL)	Normal	Ref	Ref	Ref	Ref	Ref	Ref
	Impaired	0.274*	0.187	0.469*	0.318*	0.359*	0.233*
	Diabetes	0.871*	0.667*	0.999*	0.892*	0.979*	0.778*
High-density lipoprotein-cholesterol (mg/dL)	<40	0.167	—	0.563*	0.511*	0.447*	0.401*
	≥40	Ref	Ref	Ref	Ref	Ref	Ref
Low-density lipoprotein-cholesterol (mg/dL)	<100	Ref	Ref	Ref	Ref	Ref	Ref
	100–159	−0.072	—	0.408*	0.491*	0.099	0.182
	≥160	0.135	—	1.064*	1.056*	0.434*	0.445*
Urinary proteins	−	Ref	Ref	Ref	Ref	Ref	Ref
	≥+1	0.587*	0.430*	0.282*	0.069	0.386*	0.198*

β1: adjusted for age and sex

β2: adjusted for age, sex, and significant variables in β1

*P-value < 0.10 in β1 and <0.05 in β2

**Table 3:** Risk scores of stroke, coronary heart disease, and atherosclerotic cardiovascular disease

**Risk factors**		**Stroke**	**Coronary heart disease**	**Atherosclerotic cardiovascular disease**
Men		0	13	7
Age	40–44 years	19	0	10
	45–49 years	29	11	18
	50–54 years	31	11	20
	55–59 years	40	14	25
	60–64 years	43	19	31
	65–69 years	52	22	35
	70–74 years	57	25	39
	≥75 years	63	28	42
Current smoking		9	7	7
Current alcohol drinking		0	−6	0
Blood pressure	High-normal	0	8	6
	Hypertension	10	17	16
Blood glucose	Impaired	0	5	4
	Diabetes	11	13	12
HDL-c <40 mg/dL		0	7	6
LDL-c (mg/dL)	100–159 mg/dL	0	7	0
	≥160 mg/dL	0	15	7
Urinary proteins ≥+1		7	0	3

**Table 4:** C-statistics of the risk scores after adding other risk factors

	**Stroke**	**Coronary heart diseases**	**Atherosclerotic cardiovascular disease**
Primary risk model	0.754	0.782	0.762
+Chronic kidney disease	0.755	0.782	0.762
+Hypertensive retinopathy	0.755	0.784	0.763
+ECG findings			
+Atrioventricular conduction defect	0.755	0.782	0.763
+High-amplitude R	0.756	0.786	0.765
+ST-T abnormalities	0.756	0.782	0.763
+Atrial fibrillation or flutter	0.755	0.782	0.763

**Table 5:** Incidence of cardiovascular diseases within 10 years by their risk scores

**Scores**	**Stroke, %**	**Coronary heart disease, %**	**Atherosclerotic cardiovascular disease, %**
<15	0.2	0.3	0.2
15–24	0.4	0.3	0.8
25–34	1.0	0.7	1.2
35–44	2.2	2.2	3.5
45–54	3.8	3.5	5.7
55–64	7.3	8.2	9.6
65–74	10.3	11.8	19.6
75–84	18.0	13.2	21.5
≥85	27.7	23.5	60.5

N.B. The maximum risk score is 100.

## 3. Results

Participants who developed ASCVD had higher proportions of men, older adults, obesity, current smoking, hypertension, diabetes, decreased HDL-c, increased LDL-c, and urinary proteins (Table 1).

Within 110,428 person-years (median follow-up = 16.6 years), 410 stroke events (261 cerebral infarction, 73 intracerebral hemorrhage, 35 subarachnoid hemorrhage, and 41 unspecified types), 288 CHD events (83 confirmed myocardial infarction, 93 potential myocardial infarction, 108 percutaneous coronary intervention or coronary artery bypass grafting, and 4 sudden cardiac deaths), and 527 ASCVD events were diagnosed (22 participants developed both cerebral infarction and CHD).

In the multivariable-adjusted model, age, smoking, hypertension, and diabetes were associated with stroke, CHD, and ASCVD risk. The risk of CHD and ASCVD was higher among men than women. Decreased HDL-c and increased LDL-c were associated with higher CHD and ASCVD risk. Urinary proteins were associated with higher stroke and ASCVD risk. Alcohol consumption was inversely associated with CHD risk (Table 2). P-values for sex interaction were provided (Supplementary Table 1). The risk scores of variables contributing to stroke, CHD, and ASCVD risk were described (Table 3). The C-statistic values were 0.754 for stroke, 0.782 for CHD, and 0.762 for ASCVD (Table 4). The p-values of the goodness-of-fit were 0.981 for stroke, 0.746 for CHD, and 0.326 for ASCVD (Fig. 1). The 10-year incidence of stroke, CVD, and ASCVD events was 0.2%, 0.3%, and 0.2% for scores <15, 3.8%, 3.5%, and 5.7% for scores 45–54, 10.3%, 11.8%, and 19.6% for scores 65–74, and 27.7%, 23.5%, and 60.5% for scores ≥85, respectively (Table 5). The age- and sex-adjusted associations with CKD, hypertensive retinopathy, and other ECG findings were provided (Supplementary Table 2). Adding these variables to the final risk models did not improve the C-statistics (Table 4).

## 4. Discussion

This study indicated new Suita risk models for stroke, CHD, and ASCVD using risk factors similar to those collected in the Japanese governmental health check-ups. Within a median follow-up of 16.6 years, age, smoking, hypertension, and diabetes were associated with stroke, CHD, and ASCVD risk. Men had a higher risk of developing CHD and ASCVD than women. Decreased HDL-c and increased LDL-c were associated with higher CHD and ASCVD risk. Urinary proteins were associated with higher stroke and ASCVD risk. Alcohol consumption was inversely associated with CHD risk. The three risk models performed well in terms of discrimination and calibration. We also developed three simple risk scores ranging between zero and 100 and calculated the 10-year incidence of stroke, CHD, and ASCVD per these scores for potential health education purposes.

Our results aligned with other national risk models [16–21]. For example, the Hisayama study suggested a model, including age, sex, smoking, Systolic BP (SBP), diabetes, HDL-c, and LDL-c, to predict CVD. Later, they added urinary protein and physical activity to create a new model predicting ASCVD [16, 17]. Two risk models were extracted from the Japan Public Health Center-Based Prospective Study (JPHC); one for stroke [18] and the other one for CHD [19]. Both models included age, sex, smoking, SBP, antihypertensive medication use, and diabetes. In addition, the stroke model included BMI and the CHD model included HDL-c and non-HDL-c [18, 19]. In the Japan Arteriosclerosis Longitudinal Study (JALS), age, sex, smoking, hypertension, and diabetes predicted stroke and myocardial infarction. HDL-c and non-HDL-c were associated with myocardial infarction but not stroke [20]. In the Jichi Medical School (JMS) Cohort Study, age, sex, smoking, SBP, and diabetes were associated with stroke and cerebral infarction risk [21]. Using the IQVIA Japan Claims Database, the risk factors for stroke were age and SBP, while those for CHD were age, sex, SBP, HDL-c, LDL-c, and total cholesterol [22]. The annual check-ups of Ibaraki prefecture showed that age, being underweight, smoking, SBP, hypertension medication, diabetes, HDL-c, total cholesterol, and urinary proteins were risk factors for CVD mortality [23]. A summary of the featured Japanese CVD prediction models is provided (Supplementary Table 3).

Compared to the previous Suita risk scores, the current scores showed some differences. For example, the maximum scores of previous Suita risk scores were 26 for stroke, 104 for CHD, and 65 for CVD [4–6], compared to 100 in the current risk scores, making the current risk scores easier for calculation and comparisons. Additionally, previous Suita risk scores included variables that are not regularly assessed in the Japanese governmental health check-ups, such as CKD in the Stroke and CHD risk scores, atrial fibrillation in the Stroke and CVD risk scores, and left ventricular hypertrophy in the CVD risk score [4–6]. Thus, the previous Suita risk scores could not be implemented in the Japanese governmental health check-ups. These variables were not included in the current risk scores.

Our study had several strengths, such as the prospective design, long follow-up period, representativeness of the study population for urban people in Japan, ascertaining stroke, CHD, and their risk factors using standardized methods, the long follow-up period, and the development of simple risk scores. However, this study is not without limitations. First, we assessed the risk factors during baseline only. Participants with chronic diseases might have received close monitoring which minimized their contribution to ASCVD risk. Alike, around 50% of smokers at baseline quit smoking during follow-up, suggesting that the risk of smoking in this study could be underestimated. Second, the previous Suita risk models [4–6] were internally validated; therefore, we did not repeat this process in the current study. However, none of the previous or new Suita risk models were externally validated. Third, while the age of participants ranged between 30 and 84 years, *Tokutei-Kenshin* check-ups are conducted for those between 40–74 years. However, when we limited our analysis to this age group, the risk models did not materially change (data are not shown). By including data for those under the age of 40 years and those aged 75 and over, it is possible to evaluate the risk of ASCVD in age groups not included in *Tokutei-Kenshin*. Fourth, we created a prediction model limited to specific medical check-up items. Therefore, lifestyle habits, such as physical activity and diet, were not examined. Both factors can significantly influence ASCVD risk [2, 3]. Fifth, because of the low statistical power, we could not assess the risk by subtypes of cerebral infarction (atherothrombotic, lacunar, and cardioembolic infarction) and hemorrhagic stroke (intracerebral and subarachnoid hemorrhage). Sixth, lifestyle habits that may impact ASCVD, such as diet and sleep, are not often assessed in Japanese governmental health check-ups. Thus, they were not included in our models.

In conclusion, using traditional CVD risk factors from the Suita Study, we developed new risk models to predict stroke, CHD, and ASCVD in Japanese people. Among the suggested risk factors, current smoking, hypertension, diabetes, reduced HDL-c, elevated LDL-c, and urinary proteins are modifiable, suggesting that early detection and management of these health conditions can potentially reduce the risk of ASCVD. Compared to the previous Suita models, the new models can be more practical on the national level because they include factors like those in the Japanese governmental health check-ups. Besides, the risk scores in the current models are easier to calculate, and consequently more convenient to be used for health guidance. From a preventive medical point of view, developing a risk tool that can evaluate other lifestyle habits that increase the contribution rate of the risk score is still required.

## Abbreviations

ASCVD: Atherosclerotic cardiovascular disease
BMI: Body mass index
BP: Blood pressure
CHD: Coronary heart disease
CKD: Chronic kidney disease
ECG: Electrocardiography
FBG: Fasting blood glucose
HDL-c: High-density lipoprotein-cholesterol
JALS: Japan Arteriosclerosis Longitudinal Study
JMS: Jichi Medical School
LDL-c: Low-density lipoprotein-cholesterol
NCVC: National Cerebral Cardiovascular Centre
SBP: Systolic blood pressure
WHO MONICA: World Health Organization Multinational MONItoring of Trends and Determinants in CArdiovascular Disease

## Supplementary information

**Additional file 1:** **Supplementary Table 1:** P-values for sex interaction with potential risk factors. **Supplementary Table 2:** Associations between potential risk factors (added one by one to the risk models) and cardiovascular disease risk. **Supplementary Table 3:** Summary of Japanese cardiovascular disease prediction models.

## Declarations

### Availability of data and materials

Available upon reasonable request.
