# Supplementary material for: Development of new scores for atherosclerotic cardiovascular disease using specific medical examination items: the Suita Study
Source: Environ Health Prev Med. 2023 Oct 27;28:61. doi: 10.1265/ehpm.23-00099 (PMC10613553; doi:10.1265/ehpm.23-00099)
Supplement: Supplementary file 1 — Additional file 1: Supplementary Table 1: P-values for sex interaction with potential risk factors. Supplementary Table 2: Associations between potential risk factors (added one by one to the risk models) and cardiovascular disease risk. Supplementary Table 3: Summary of Japanese cardiovascular disease prediction models. [file ehpm-28-061-s001.docx]

**Supplementary Table 1: P-values for sex interaction with potential risk factors**

| Risk factors | Stroke | Coronary heart disease | Atherosclerotic cardiovascular disease |
| --- | --- | --- | --- |
| Age | 0.466 | 0.226 | 0.036 |
| Body mass index | 0.770 | 0.303 | 0.591 |
| Waist circumference | 0.385 | 0.998 | 0.325 |
| Smoking | 0.502 | 0.175 | 0.529 |
| Alcohol drinking | 0.495 | 0.465 | 0.110 |
| Blood pressure | 0.526 | 0.119 | 0.274 |
| Blood glucose | 0.892 | 0.386 | 0.715 |
| High-density lipoprotein-cholesterol | 0.249 | 0.542 | 0.364 |
| Low-density lipoprotein-cholesterol | 0.608 | 0.227 | 0.158 |
| Urinary proteins | 0.141 | 0.049 | 0.155 |

**Supplementary Table 2: Associations between potential risk factors (added one by one to the risk models) and cardiovascular disease risk**

| Potential risk factors not included in the primary risk models | | Stroke, ß | Coronary heart diseases, ß | Atherosclerotic cardiovascular disease, ß |
| --- | --- | --- | --- | --- |
| + Chronic kidney disease | No | Ref | Ref | Ref |
|  | Yes | 0.439* | 0.311* | 0.246* |
| + Hypertensive retinopathy | No | Ref | Ref | Ref |
|  | Yes | 0.442* | 0.743* | 0.598* |
| + Atrioventricular conduction defect | No | Ref | Ref | Ref |
|  | Yes | 0.878* | 0.716* | 0.967* |
| + High-amplitude R | No | Ref | Ref | Ref |
|  | Yes | 0.421* | 0.376* | 0.482* |
| + ST-T abnormalities | No | Ref | Ref | Ref |
|  | Yes | 0.723* | 0.691* | 0.778* |
| + Atrial fibrillation or flutter | No | Ref | Ref | Ref |
|  | Yes | 0.749* | 0.944* | 0.873* |

ß: adjusted for age and sex

*P-value < 0.10

**Supplementary Table 3: Summary of Japanese cardiovascular disease prediction models**

| Study | Population | Outcome | Risk factors |
| --- | --- | --- | --- |
| Arafa et al. [4] | 6,641 from the Suita Study (30-79 years) | Stroke | Age, smoking, hypertension, diabetes, CKD, and AF |
| Nishimura et al. [5] | 5,512 from the Suita Study (30-79 years) | Coronary heart disease | Age, sex, smoking, hypertension, diabetes, TC, HDL-c, and CKD |
| Nakai et al. [6] | 6,550 from the Suita Study (30-79 years) | Cardiovascular disease | Age, sex, smoking, hypertension, diabetes, non-HDL-c, HDL-c, urinary protein, AF, and LVH |
| Arima et al. [16] | 2,634 from the Hisayama Study (≥ 40 years) | Cardiovascular disease | Age, sex, smoking, hypertension, diabetes, LDL-c, and HDL-c |
| Honda et al. [17] | 2,454 from the Hisayama Study (40-84 years) | Atherosclerotic cardiovascular disease | Age, sex, smoking, hypertension, diabetes, LDL-c, HDL-c, proteinuria, and exercise |
| Yatsuya et al. [18] | 15,672 from the JPHC (40-69 years) | Stroke | Age, sex, smoking, BMI, hypertension, antihypertensive medication, and diabetes |
| Yatsuya et al. [19] | 15,672 from the JPHC (40-69 years) | Coronary heart disease and ischemic stroke | Age, sex, smoking, BMI, hypertension, antihypertensive medication, diabetes, and HDL-c |
| Harada et al. [20] | 67,969 from the JALS (40-89 years) | Cardiovascular disease, stroke, and myocardial infarction | Age, sex, smoking, hypertension, and diabetes in all models. HDL-c in myocardial infarction and cardiovascular disease. Non-HDL-c in myocardial infarction |
| Ishikawa et al. [21] | 12,276 from the JMS Cohort Study (19-93 years) | Stroke | Age, sex, smoking, hypertension, and diabetes |
| Yoshida et al. [22] | 572,971 from the IQVIA Japan Claims Database | Stroke and coronary heart disease | Age and hypertension in both models. Sex, HDL-c, LDL-c, and TC in coronary heart disease |
| Noda et al. [23] | 92,275 from the annual check-ups of Ibaraki prefecture (40-97 years) | Cardiovascular disease, stroke, and coronary heart disease mortality (Sex-specified) | Coronary heart disease: Age, smoking, BMI, hypertension, antihypertensive medication, diabetes, TC, HDL-c, creatinine, and urinary protein. Stroke: age, BMI, smoking, hypertension, antihypertensive medication, HDL-c, creatinine, and AST or ALT. Coronary heart disease: Age, smoking, hypertension, diabetes, TC, HDL-c, and urinary protein among men |

AF: Atrial fibrillation; ALT: Alanine transaminase, AST: Aspartate aminotransferase, BMI: Body mass index; CKD: Chronic kidney disease; HDL-c: High-density-lipoprotein-cholesterol; JALS: Japan Arteriosclerosis Longitudinal Study; JPHC: The Japan Public Health Center-based prospective study; JMS: Jichi Medical School; LDL-c: Low-density-lipoprotein-cholesterol; LVH: Left ventricular hypertrophy; TC: Total cholesterol
